# Systemic sclerosis-associated severe gastric antral vascular ectasia treated with tocilizumab:A case report and review of the literature

**DOI:** 10.1177/23971983241309570

**Published:** 2025-01-08

**Authors:** Stefano Rodolfi, Christopher P Denton, Voon H Ong

**Affiliations:** University College London Medical School, London, UK

**Keywords:** Tocilizumab, gastric antral vascular ectasia, immunosuppression for gastrointestinal manifestations, systemic sclerosis, RNA polymerase III

## Abstract

Gastric antral vascular ectasia is a frequent and potentially severe complication of systemic sclerosis. Management is presently limited to supportive care, acid suppression and endoscopic treatment. Many cases of gastric antral vascular ectasia tend to be refractory or partially responsive to standard treatment and require multiple endoscopic procedures to control the recurrent bleeding. Immunosuppression is not part of the recommended management of gastric antral vascular ectasia: limited data exist on the role of cyclophosphamide or autologous stem cell transplant in severe cases, but no prospective data or randomised controlled trial supports its routine use. Here, we present a case of an adult male patient with diffuse cutaneous systemic sclerosis complicated by arthritis and severe gastric antral vascular ectasia. The latter required multiple endoscopic procedures and remained transfusion-dependent. Due to progressive skin disease and active arthritis refractory to conventional synthetic disease-modifying antirheumatic drugs, the patient was started on tocilizumab. While he showed an early response in terms of scores related to skin involvement and arthritis, response to gastric antral vascular ectasia was unexpected. As soon as the biologic therapy was started, the patient was no longer transfusion-dependent and haemoglobin levels started to rise. Subsequent endoscopic investigations confirmed resolution of gastric antral vascular ectasia. This case is illustrative of an unexpected response to tocilizumab, and this observation is supported by the biological rationale of interleukin-6 in vascular remodelling.

## Case description

The patient, a 62-year-old man, was referred to our tertiary centre due to a recent diagnosis of anti-RNA polymerase III-positive diffuse cutaneous systemic sclerosis (SSc) with arthritis and transfusion-dependent gastric antral vascular ectasia (GAVE). Symptoms started 1 year prior to the referral, with progressive skin puffiness and subsequent thickening, involving proximal extension of both upper and lower limbs. He also reported progressive fatigue due to iron deficiency anaemia, for which he underwent an oesophagogastroduodenoscopy (OGD) which documented GAVE, and a capsule endoscopy which showed multiple distal small bowel bleeding sites. Prior to his attendance at our centre, he had undergone multiple endoscopic treatments with argon plasma coagulation (APC), with poor efficacy, and was requiring blood transfusions close to weekly intervals. Complicating the clinical scenario, the patient had a history of ischaemic heart disease, managed with coronary artery bypass grafting 3 years prior to GAVE onset with two re-stenting procedures. He remained on dual anti-platelet therapy with aspirin and clopidogrel, and he was experiencing recurrent angina episodes with anaemia.

**Figure 1.**
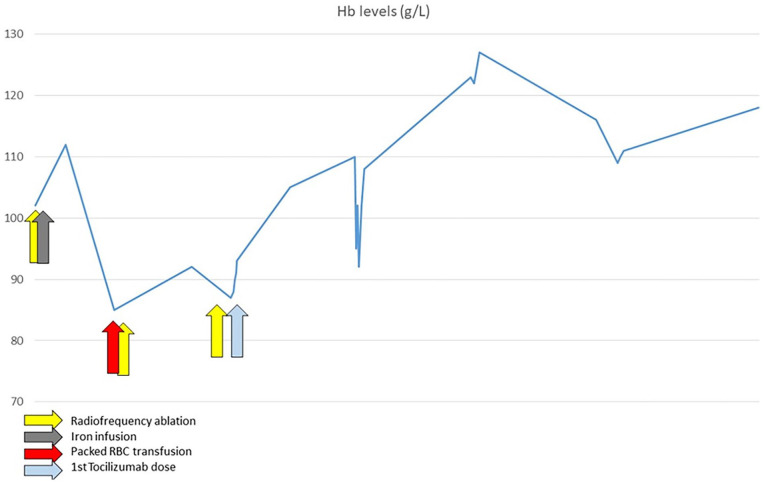
Trajectory of haemoglobin in response to therapeutic procedures. It can be noted that since starting therapy with tocilizumab, Hb gradually rose without the need for further endoscopic procedures or blood transfusions.

**Figure 2.**
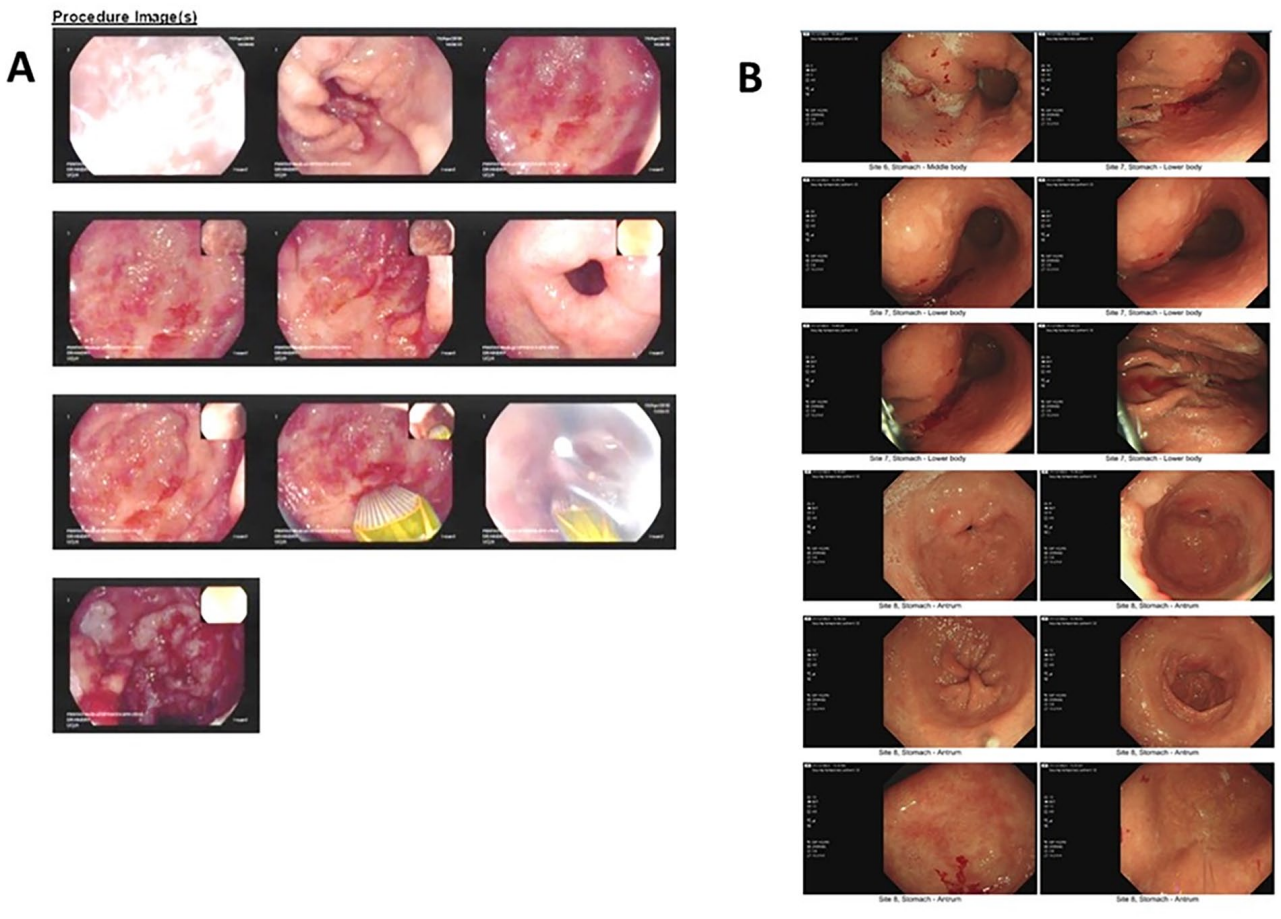
Panel A shows gastric antral region during an APC procedure: multiple radiating stripes of vessels can be noted, in a typical GAVE picture. Panel B shows the last OGD, undergone after 4 years on tocilizumab therapy: mild erythema is documented in gastric antrum, but no signs of GAVE.

On initial review in our centre, he required fortnightly blood transfusions. He did not have any evidence of interstitial lung disease (ILD) nor myositis, while his blood showed anaemia with moderately elevated inflammatory markers (white blood cell (WBC) count 10.32 10^9^/L of which 77% neutrophils, C-reactive protein (CRP) 14 mg/L) and were otherwise unremarkable. He was started on mycophenolate mofetil (MMF) 2 g/day for skin disease unresponsive to methotrexate 25 mg/week, and we referred him to have radiofrequency ablation (RFA) treatment for GAVE. Over the subsequent 5 months, he was subjected to three RFAs without significant improvement and continued with frequent blood transfusions. He was considered to be at high anaesthetic risk for subtotal gastrectomy. Further plans for repeat RFA were abandoned, as he was deemed unfit for this procedure. Anti-platelet therapies continued during this period. At this point, as his arthritis was floridly active with a disease activity score-28 (DAS-28) of 6.1 and skin involvement was progressing despite treatment with MMF, he was commenced on weekly subcutaneous tocilizumab 162 mg. Three weeks after starting tocilizumab, modified Rodnan’s skin score stabilised at 34/51 with a reduction of DAS-28 down to 4.1. Remarkably, he did not require further blood transfusions with improvement in Hb and reduction in anginal frequency (Figure 1). The patient has remained on tocilizumab for 6 years now and has not required any blood transfusion or endoscopic procedures. Figure 2 depicts the different endoscopic appearance of gastric mucosa before tocilizumab and after 4 years on treatment.

## Discussion

GAVE is not an infrequent complication in SSc, with a reported prevalence ranging between 1% and 10.6%.^1, 2^ However, this prevalence is likely underestimated and reflects clinical practice, where clinical GAVE is usually diagnosed with endoscopic assessment. A higher prevalence of subclinical GAVE, up to 34.6%, was reported in screening studies with OGD or video-capsule endoscopic evaluation.^3^ GAVE has been associated with the presence of cutaneous telangiectasias, a higher nailfold video-capillaroscopy score and the presence of anti-RNA polymerase III antibody, while conflicting evidence has been reported on the association with the cutaneous subset. Pathogenesis of GAVE in SSc is incompletely understood, and its clinical characteristics may indicate shared pathogenetic mechanisms with hereditary haemorrhagic telangiectasia, an autosomal dominant disorder of the vasculature development characterised by telangiectasia and arteriovenous malformations affecting the gut in up to 30% of cases.^4^ An alternative proposed explanation is that both disordered gastric muscular motility and antral mucosal prolapse through the pylorus, the latter caused by a loose connection between the distal gastric mucosa and the adjacent muscularis externa, concur in causing submucosal ischaemia and both elongation and dilatation of mucosal vessels. This hypothesis is strengthened by the documented histological features of fibromuscular hyperplasia of the lamina propria and dilatation of the mucosal capillaries. The presence of uncoordinated high-amplitude gastric antral contractions on manometric studies lends further support to this hypothesis.^5^ There is no clear-cut evidence on the role of immune dysfunction. A recent report on gastric biopsies in patients with SSc identified the presence of a prominent CD4+ T lymphocyte infiltrates with markers of lymphocyte activation.^6^ However, none of the patients in this study had GAVE. Indirect evidence on the role of immune dysregulation in the pathophysiology of GAVE comes from anecdotal reports of potential benefit of immunosuppressive strategies on this manifestation.

Management of GAVE depends on clinical severity. Mild cases can be managed with supportive care, including iron supplementation (oral or intravenous), proton pump inhibitors, correction of possible underlying coagulopathy and avoiding drugs that might exacerbate the condition, such as anti-platelet agents or non–steroidal anti-inflammatory drugs (NSAIDs). In the event of significant anaemia, transfusions of packed red blood cells and/or endoscopic treatment may be needed. Clinically significant bleeding or anaemia refractory to supportive care prompts for endoscopic treatment. To date, no randomised controlled trial exists to favour one endoscopic technique over another, but APC represents the most common approach in recent years.^7^ APC is associated with a high success rate with resolution of anaemia; however, the recurrence of GAVE of over 50% at 1 year has been reported. RFA can be used as an alternative to or second line after APC. RFA is associated with similar success rates of APC; however, most are referred to RFA if they failed APC, and the fact that RFA is able to treat a larger mucosal area might suggest that RFA is a more effective endoscopic technique in treating GAVE.^7^ Surgery with antrectomy or gastrectomy is reserved to patients fit for surgery with severe GAVE resistant to medical and endoscopic therapy.

The role of immunosuppression has been controversial in GAVE. A role for autoimmunity has been hypothesised based on the correlation with autoantibody subtypes and the observed response to immunosuppressive treatment. The evidence is, however, limited to case reports and small case series and largely restricted to cyclophosphamide. The first report dates to 2001, when Lorenzi et al. reported the case of a patient with SSc-associated severe GAVE who was treated with intravenous (IV) methylprednisolone and cyclophosphamide for progressive ILD and diffuse skin disease; together with improvement in ILD and cutaneous involvement, his Hb levels stabilised and subsequent OGDs documented resolution of GAVE. Schultz et al. described the records of three patients with severe GAVE treated with IV pulses of cyclophosphamide (one in the context of chemotherapy for lymphoma). All of them showed a remarkable clinical and endoscopic improvement of GAVE, without the need for additional transfusions and, in 2/3 of cases, for further endoscopic treatment. Papachristos et al. reported the cases of two patients with severe GAVE successfully treated with IV cyclophosphamide, which was started specifically for GAVE. In addition, Matsumoto et al.^8^ reported a remarkable clinical and endoscopic response of GAVE to IV cyclophosphamide in one patient, once again started specifically for the treatment of GAVE. A recent large retrospective cohort study, despite not having undergone to address treatment, did not show any benefit for immunosuppressive therapy including cyclophosphamide in GAVE.^2^ Bhattacharyya et al. reported improvement of GAVE following haematopoietic stem cell transplant (HSCT) in three patients, who received it for severe skin and lung disease refractory to cyclophosphamide. All of them achieved transfusion independence by Day 20 post-HSCT, gained almost 50 g/L of Hb and maintained stable Hb levels throughout the next 12 months.^9^

To our knowledge, this is the first reported case of GAVE treated with tocilizumab. Tocilizumab is a humanised IgG1 class monoclonal antibody targeting the interleukin-6 receptor (IL-6R) and preventing its binding to interleukin-6 (IL-6). IL-6 has a well-established role in mediating skin and lung fibrosis in SSc, and treatment with tocilizumab has provided to be beneficial for SSc-ILD. However, the role of IL-6 in mediating SSc vasculopathy, and especially gastrointestinal (GI) vasculopathy, is not defined yet. Notably, tocilizumab, despite its effect on hyperlipidaemia, paradoxically improved endothelial function in a patient cohort with high cardiovascular risk.^10^ The remarkable long-standing clinical and endoscopic response of GAVE in the presented case was undoubtedly linked to tocilizumab. This serendipitous observation may provide insights into novel biological pathways relevant to GAVE pathophysiology and to alternative therapeutic options which may significantly impact a disease manifestation which still bears a significant morbidity and mortality.

## Conclusion

This is, to our knowledge, the first reported case of severe, refractory GAVE treated with tocilizumab. The response to IL-6 inhibition was remarkable and unequivocally temporally linked with the initiation of tocilizumab, in that the patient, previously transfusion-dependent, did not require subsequent blood transfusion nor endoscopic procedures.
